# Characterization of *Brucella canis* infection in mice

**DOI:** 10.1371/journal.pone.0218809

**Published:** 2019-06-20

**Authors:** Lauren W. Stranahan, Omar H. Khalaf, Daniel G. Garcia-Gonzalez, Angela M. Arenas-Gamboa

**Affiliations:** 1 Department of Veterinary Pathobiology, Texas A&M University, College of Veterinary Medicine, College Station, TX, United States of America; 2 Department of Veterinary Pathology & Poultry Diseases, College of Veterinary Medicine, University of Baghdad, Baghdad, Iraq; East Carolina University Brody School of Medicine, UNITED STATES

## Abstract

Canine brucellosis, caused by *Brucella canis*, is a disease of dogs and represents a public health concern as it can be transmitted to humans. Canine brucellosis is on the rise in the United States and there is currently no vaccine for use in dogs. Mice have been extensively utilized to investigate host-pathogen interactions and vaccine candidates for smooth *Brucella* species and could serve a similar role for studying *B*. *canis*. However, comparatively little is known about *B*. *canis* infection in mice. The objective of this study was to characterize the kinetics of colonization and pathogenicity of *B*. *canis* in mice in order to evaluate the mouse as a model for studying this pathogen. C57BL/6 mice were inoculated intraperitoneally with 10^5^, 10^7^, or 10^9^ CFU of *Brucella canis* RM6/66 and euthanized 1-, 2-, 4-, 6-, 9-, and 12-weeks post-inoculation. *B*. *canis* induced splenomegaly in mice infected with 10^9^ CFU at 1- and 2 weeks post-inoculation while no gross lesions were observed in other dose groups. Infection at the two higher doses resulted in dose-dependent granulomatous hepatitis and histiocytic infiltration of the spleen and mesenteric lymph nodes by 1–2 weeks. *B*. *canis* was cultured from the liver, spleen, uterus, bone marrow, lung, and kidney in all groups with colonization declining at a slow but steady rate throughout the experiment. Clearance was achieved by 9 weeks 10^5^ CFU group and by 12 weeks in the 10^7^ CFU group, while *B*. *canis* persisted in the spleen until 12 weeks in the highest dose group. Although *B*. *canis* does not demonstrate significant replication in C57BL/6 mice, it has the ability to establish an infection, induce splenomegaly, and persist for several weeks in multiple organs. Moreover, 1 x 10^7^ CFU appears to be a suitable challenge dose for investigating vaccine safety.

## Introduction

Brucellosis is a disease of animals and humans caused by Gram negative, facultatively intracellular bacteria of the genus *Brucella*. Of the 12 currently recognized species, four are considered zoonotic pathogens: *B*. *melitensis*, *B*. *suis*, *B*. *abortus*, and *B*. *canis* in decreasing order of pathogenicity. Canine brucellosis, caused by *B*. *canis*, is a worldwide disease of dogs that primarily results in reproductive disease and may be transmitted to humans [1,2]. In bitches, this manifests as abortion and in males, predominant symptoms include prostatitis and epididymitis [3,4]. Like all *Brucella* species, *B*. *canis* invades via the conjunctival, oronasal, or venereal route and distributes to organs of the reticuloendothelial system, resulting in a chronic, persistent infection [5]. Clinical signs may not become apparent in infected dogs for months to years after infection, making it difficult to implement control measures and avoid spread of disease to other dogs and humans [6,7].

While canine brucellosis has historically been considered an important pathogen of kenneled dogs, *B*. *canis* is now isolated with increasing frequency from shelter and stray dog populations throughout the world [1,8]. Of greater concern is the similarly rising number of reported cases of human infection [9–11]. While *B*. *canis* is perceived to be less virulent, manifestation of human disease can occasionally resemble that induced by smooth *Brucella* species [12,13]. Methods of control are currently limited to screening for infection in dogs, which relies predominantly on a complex array of serologic tests, none of which provide ideal sensitivity or specificity [5,14]. Unfortunately, while vaccination remains a cornerstone of brucellosis control for the smooth strains (i.e., *B*. *melitensis*, *B*. *abortus*, and *B*. *suis*), no vaccine is currently available to protect against the rough strain, *B*. *canis* [15].

Mice have become the most frequently employed animal model for studying brucellosis due to greater economic and ethical constraints associated with studies involving natural hosts, particularly livestock [16]. While results obtained from mice are not immediately transferable to humans or natural animal hosts due to differences in immune response and susceptibility to infection, the advent of mutant and transgenic mice has allowed mice to become invaluable tools for elucidating host-pathogen interactions and assessing efficacy of vaccine candidates. While the behavior of smooth strains of *Brucella* has been extensively studied in numerous mouse strains, little work has been done to characterize *B*. *canis* infection in this valuable animal model. Most importantly, without this understanding, protocols including challenge dose utilized in vaccine efficacy studies cannot be extrapolated from previous work involving smooth *Brucella* strains.

In the present study, we describe a mouse model for investigating the pathogenesis of *Brucella canis* infection and vaccine efficacy against this pathogen, including kinetics of dissemination following intraperitoneal injection, macroscopic and microscopic lesions, and humoral immune response. Just as previous studies have indicated that mice are a valuable tool for studying host-pathogen interactions and assessing vaccine safety and efficacy for smooth strains of *Brucella*, we propose that mice could prove useful in this manner in regards to rough strains such as *B*. *canis*.

## Materials and methods

### Ethics statement

This study was carried out in an approved facility in strict accordance with all university and federal regulations. All mouse experimentation was reviewed and approved by the Texas A&M University Laboratory Animal Care and Use Committee (protocol: 2018–0046). The protocol was approved and is in accordance with the Institutional Animal Care and Use Committee (IACUC) policies of Texas A&M University. Texas A&M is accredited by the Association for the Assessment and Accreditation of Laboratory Animal Care, International (AAALAC).

### Animals

Female, 6–8 week old, C57BL/6 mice (n = 96) were obtained from the Texas A&M Institute for Genomic Medicine and housed in microisolater caging in a biosafety level 3 facility. Mice were acclimated to the facility for 5 days prior to infection and were maintained on a 12-hour—12-hour light-dark cycle with ad libitum access to pelleted food and water. Mice were monitored daily for signs of pain or distress according to the guidelines of the Animal Research Advisory Committee published by the National Institutes of Health.

### Bacterial strains

*B*. *canis* ATCC RM6/66 was used for these studies. Bacteria were stored at -80°C in 10% glycerol and were routinely grown on tryptic soy agar (TSA) plates or in standard tryptic soy broth (TSB).

### Kinetics of infection

Mice were randomly divided into three experimental groups (*n* = 30) and one control group (*n* = 6). Each experimental group was inoculated intraperitoneally (i.p.) with 1x10^5^, 1x10^7^, or 1x10^9^ colony forming units (CFU) of *B*. *canis* RM-6/66 while the control group was inoculated with phosphate-buffered saline (PBS). Mice were sacrificed by CO_2_ asphyxiation followed by cervical dislocation at 1-, 2-, 4-, 6-, 9-, and 12-weeks post-inoculation. At each time point, samples of liver, spleen, uterus, lung, kidney, and bone marrow were aseptically collected into 1 ml PBS, homogenized, serially diluted, and 100 μl of each dilution was plated in duplicate onto Farrell’s medium (TSA plus Brucella 391 Oxoid supplement, equine serum, and 50% dextrose) and incubated at 37°C in an atmosphere containing 5% (vol/vol) CO_2_. Bacterial colonies were enumerated after 72 hours to quantify tissue colonization. Levels of infection were expressed as mean values and standard deviations SDs (n = 5) of the log number of CFU per gram of tissue. Spleen and liver were weighed at necropsy, and the aforementioned tissues in addition to mesenteric lymph nodes and heart were collected and fixed in 10% neutral buffered formalin for routine histopathologic evaluation.

### Histopathology

Spleen, liver, uterus, lung, heart, kidney, and mesenteric lymph nodes were collected at necropsy and fixed in 10% neutral buffered formalin for a minimum of 48 h. Tissues were routinely processed and embedded, sectioned at 5 μm, and stained with hematoxylin and eosin. Sections from spleen, mesenteric lymph nodes, and liver were graded in a blinded fashion (LS) on a scale of 0–4 for inflammation type and severity (S1 Table). The mean total score for each tissue was compared between groups at each time point.

### Immunohistochemistry

Unstained slides from liver, spleen, uterus, and mesenteric lymph nodes were adhered to positively charged glass slides for immunohistochemistry. Slides were deparaffinized and rehydrated through a series of xylene and ethanol steps before antigen retrieval was performed using 1:10 EMS Solution A (Electron Microscopy Services) in a 2100 Antigen Retriever (Aptum Biologics Ltd.), according to the manufacturer’s instruction. Endogenous peroxidase activity was blocked by 10 m incubation with Bloxall Blocking Solution (Vector Laboratories) followed by 20 m blocking with normal goat serum (Vector). After each step slides were washed with PBS plus 0.5% tween for 5 minutes. Primary incubation was overnight at 4°C with *Brucella* polyclonal rabbit antibody (Bioss) at 1:400. Negative control tissues were incubated with rabbit nonimmune serum diluted in PBS. A Vectastain ABC and Betazoid DAB chromagen kits (Biocare Medical) were used following primary incubation according to the manufacturer’s instructions. The slides were counterstained with Meyer’s hematoxylin III.

### Anti-*Brucella* specific total IgG ELISA

Mice were bled by ventral tail vein puncture prior to infection and prior to sacrifice at each time point. Blood was centrifuged at 3000 rpm for 5 minutes and serum was collected for anti-*B*. *canis* specific immunoglobulin G (IgG) indirect enzyme linked immunosorbent assay (iELISA). Briefly, 96-well plates (Costar, Corning, NY, USA) were coated with 500 ng/well of *B*. *canis* RM6/66 heat-killed lysate in coating buffer (pH 9.6, 0.05 M carbonate buffer) at 4°C overnight. Plates were washed three times with PBS containing 0.05% Tween 20 (PBST) and nonspecific binding was blocked with 100 μL of 0.25% bovine serum albumin (BSA) in PBS at 37°C for 2 h. Following three washes, sera diluted at 1:500 were added and incubated at 37°C for 1 h. Plates were washed five times and HRP-labeled goat anti-mouse IgG (1:1000, Vector Laboratories) was added, incubated at 37°C for 1 h. Afterwards, OPD peroxidase substrate was added (100 μL/well) and incubated for 30 minutes at 37°C in the dark. The enzyme reaction was stopped by addition of 0.5M NaOH and absorbance was measured at 450 nm. All assays were performed in triplicate, and the results are presented as the mean value for the three wells.

### Statistical analysis of data

Analysis was performed using GraphPad Prism software, version 6.0, San Diego, CA. The CFU data were normalized by log transformation and evaluated by two-way analysis of variance (ANOVA) repeated-measures test. Tukey’s multiple comparisons test was used to generate P values for mean comparisons. Splenic weight and histologic scores were compared using two-way ANOVA and Tukey’s multiple comparisons test was used to generate P values. In all analyses, a P value less than 0.05 constituted statistical significance.

## Results

### *Brucella canis* causes a dose-dependent, systemic infection in mice

**Fig 1:**
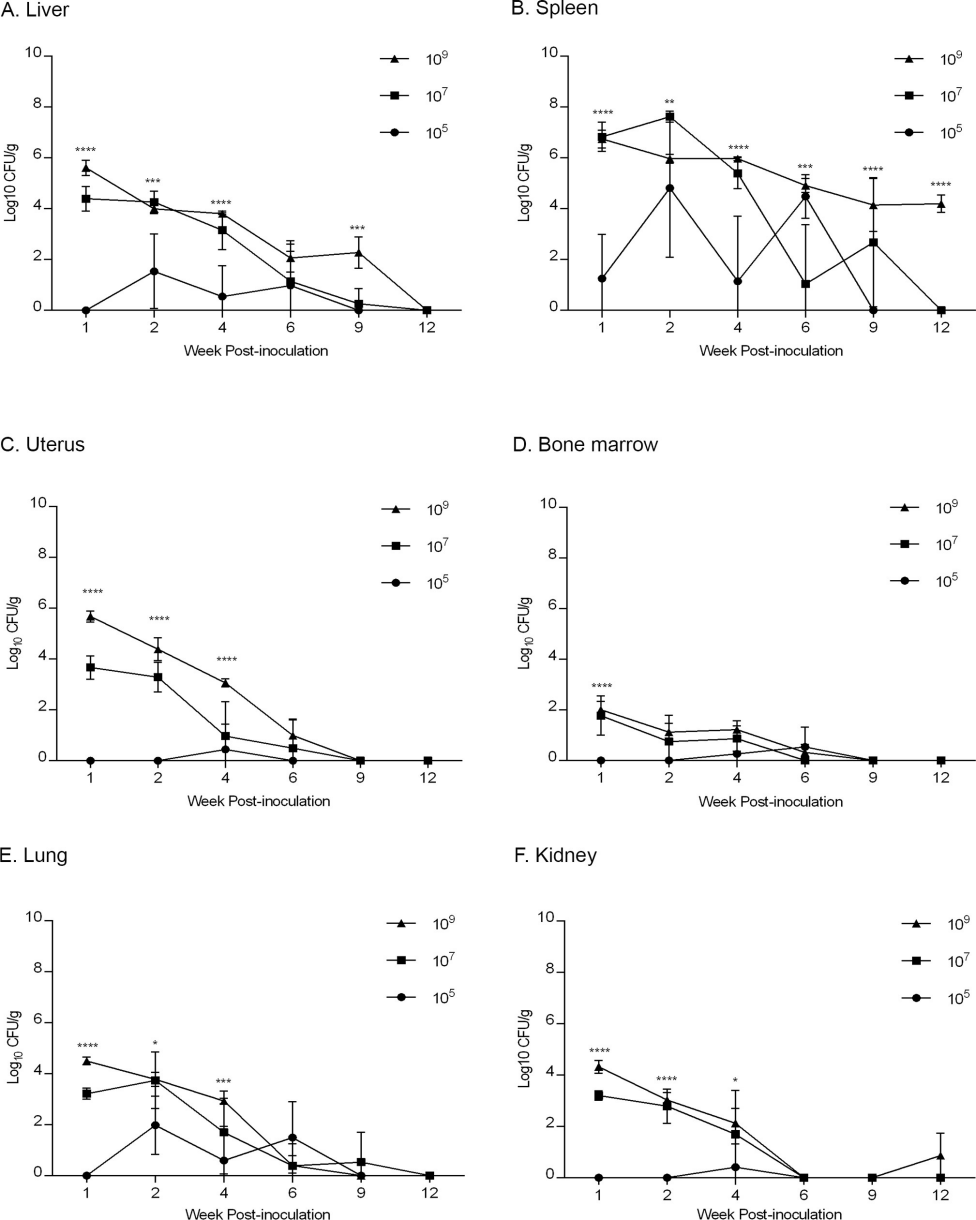
Kinetics of bacterial colonization of *B*. *canis* RM6/66 in mice. Animals were divided into 4 groups (n = 30) consisting of lower dose (10^5^, 10^7^), high dose (10^9^), or control (PBS) groups. Animals were inoculated i.p. and 5 animals from each group were euthanized at 1-, 2-, 4-, 6-, 9-, and 12-weeks post-infection. Colonization was evaluated in the liver (A), spleen (B), uterus (C), bone marrow (D), lung (E), and kidney (F). Bacterial recovery is displayed as the total CFU/g. Data points represent the mean recovery per gram of tissue plus the standard deviation for all animals in each dose group at each time point. Statistical significance was determined by ANOVA followed by Tukey’s multiple comparisons test. One asterisk, P <0 0.05. Two asterisks, P < 0.01. Three asterisks, P < 0.0001.

*Brucella* spp. preferentially colonize and establish a persistent infection in organs of the reticuloendothelial system and reproductive tract. *B*. *canis* also commonly results in intermittent bacteremia in infected dogs that may last for years after infection [8]. To investigate organ colonization, distribution, and possible locations of tropism following *B*. *canis* infection in mice, the spleen, liver, uterus, lung, kidney, and bone marrow were collected for culture. Infection at all dose groups (10^5^, 10^7^, 10^9^) resulted in colonization of all organs examined in a dose-dependent manner. The mean CFU recovered per gram of tissue was significantly higher in the high dose group (10^9^) compared with the lower dose groups (10^5^, 10^7^) at week-1 post-infection in the liver, uterus, lung, and kidney and with the lowest dose group (10^5^) at 2- and 4-weeks post-infection in the liver, spleen, and uterus (Fig 1A–1F).

Interestingly, no significant increase in mean CFU, indicative of replication, was noted in any dose group as the experiment progressed. Instead, colonization progressively declined in all tissues until clearance was achieved in all organs apart from the spleen by 9-weeks post-infection in the lower dose groups (10^5^, 10^7^). At 1-week post-infection, *B*. *canis* colonized the spleen at approximately 7 logs (6.8x10^6^ CFU/g) in the higher dose groups (10^7^, 10^9^) while the level of colonization was significantly lower in the low dose group (10^5^) at approximately 1 log, or 18 CFU/g (Fig 1B). Thereafter, colonization in the mid dose group (10^7^) maintained a steady level until a precipitous drop at 6-weeks post-infection followed by clearance at 12 weeks. Splenic colonization in the low dose group (10^5^) fluctuated widely with an inconsistent number of animals exhibiting colonization during the first 6-weeks post-infection (S2 Table). Splenic colonization in this group never exceeded 5 logs with complete clearance achieved by 9-weeks post-infection. While no colonization was noted in the spleen of the lower dose groups (10^5^, 10^7^) at 12-weeks post-infection, persistence of *B*. *canis* was noted in the high dose group (10^9^) which maintained a level of colonization of approximately 4 logs, or 1.6x10^4^ CFU/g (Fig 1B). A similar pattern was observed in the liver and uterus, in which colonization at 1-week post-infection was approximately 6 logs and 4 logs in the high (10^9^) and mid (10^7^) dose groups, respectively while colonization in the low dose group (10^5^) remained low, never exceeding 2 logs with clearance achieved in the liver by 9-weeks and in the uterus by 6-weeks post-infection (Fig 1A and 1C). Colonization in the lung and kidney was similar between dose groups with approximately 4 logs and 3 logs achieved in the high (10^9^) and mid (10^7^) dose groups by 1-week post-infection, values that were 1–2 logs lower than that observed in the liver and uterus (Fig 1E and 1F). Colonization of the lung and kidney by the low dose group again was low, never exceeding 2 logs. In all dose groups, colonization was lowest in the bone marrow with values never exceeding 2 logs and complete clearance in all dose groups achieved by 9-weeks post-infection (Fig 1D).

### Mice infected with *B*. *canis* develop macroscopic and microscopic lesions resembling natural infection

**Fig 2:**
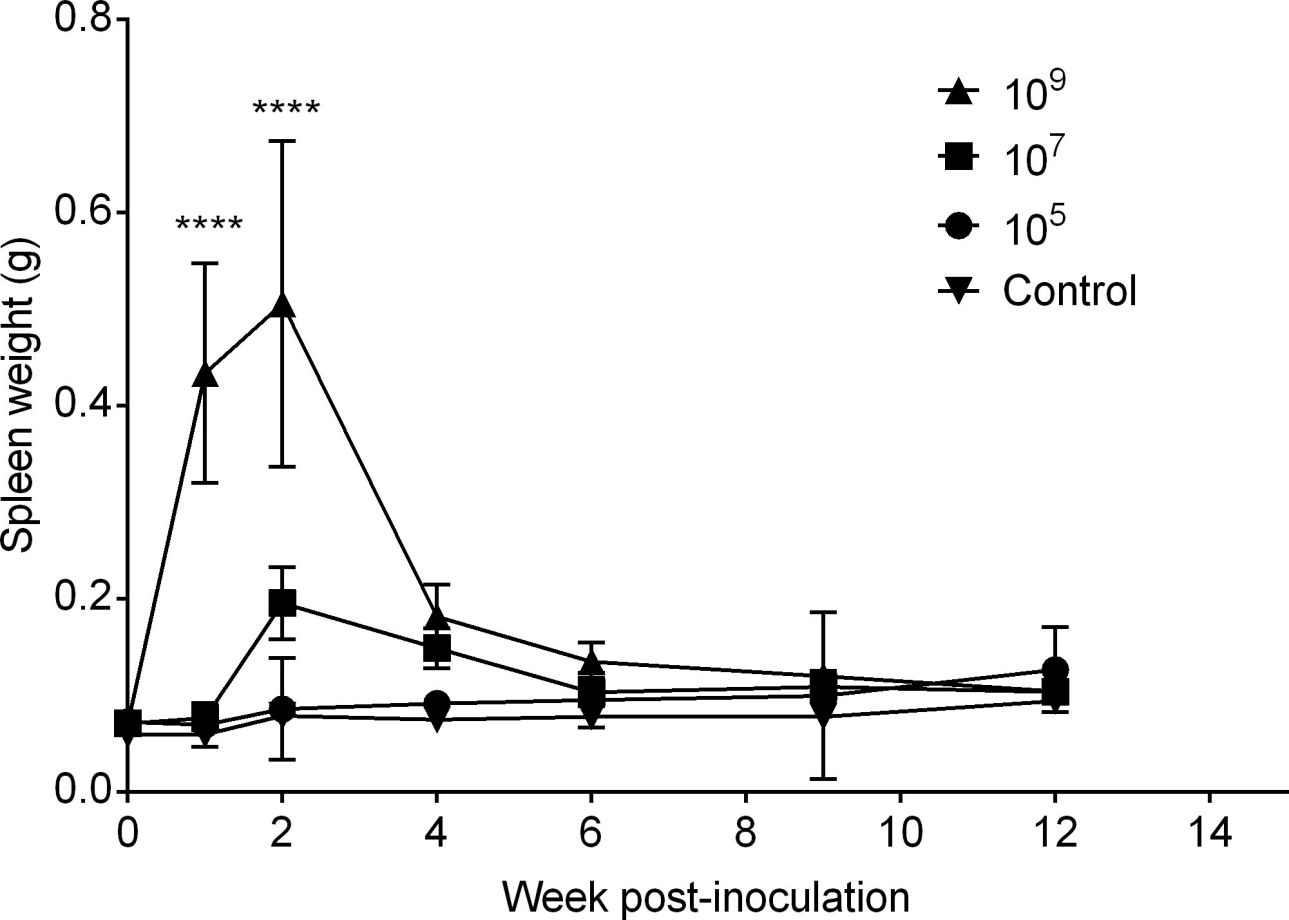
Splenic weight in mice inoculated with *B*. *canis* RM6/66 or PBS over 12 weeks. Splenomegaly was induced by a high dose (10^9^) of *B*. *canis* by 1-week post-infection (A). Splenic weight peaked at 2-weeks post-infection and then declined with no significant differences noted between treatment groups and the control group by 6-weeks post-infection. Data points represent the mean spleen weight plus the standard deviation for all animals in each dose group at each time point. Mean spleen weight from each dose group (n = 5) was compared at each time point to mean spleen weight of the uninfected control mice (n = 6) and statistical significance was determined by ANOVA followed by Tukey’s multiple comparison test. Three asterisks, P <0.0001.

Brucellosis in natural hosts and humans classically results in splenomegaly, a change that is routinely seen in mice infected with smooth *Brucella* strains at inoculating doses as low as 10^5^ CFU [16]. In response to infection, spleen weight was significantly increased (p<0.001) in the high dose group (10^9^) compared with the lower dose groups (10^5^, 10^7^) and to the uninfected controls (Fig 2). This effect was transient, with significant splenomegaly noted at 1-week post-infection, peaking at 2-weeks post-infection, and thereafter precipitously declining until no significant differences in spleen weight were noted between groups by 6-weeks post-infection (Fig 2). The average splenic weight at 2-weeks post-inoculation in the 10^5^, 10^7^, and 10^9^ group was 0.086 g, 0.196 g, and 0.506 g, respectively, compared to 0.079 g in the control group. While the liver can be colonized by *B*. *canis* in dogs and humans and can induce liver enlargement in humans [10], hepatomegaly is not associated with infection in dogs [17]. In the high dose group (10^9^), the liver weight was significantly higher than the other groups (S1 Fig). Thereafter, no significant differences in liver weight were noted between dose groups. In addition to a transient increase in liver size, macroscopic changes were detected in the high dose group (10^9^) at 1- and 2-weeks post-infection characterized by a loss of the normal tan, homogeneous appearance and replacement by multifocal zones of pallor surrounded by a thin hyperemic zone (S2 Fig). No macroscopic lesions were observed in any organ in the PBS control or lower dose groups (10^5^, 10^7^) at any time point.

**Fig 3:**
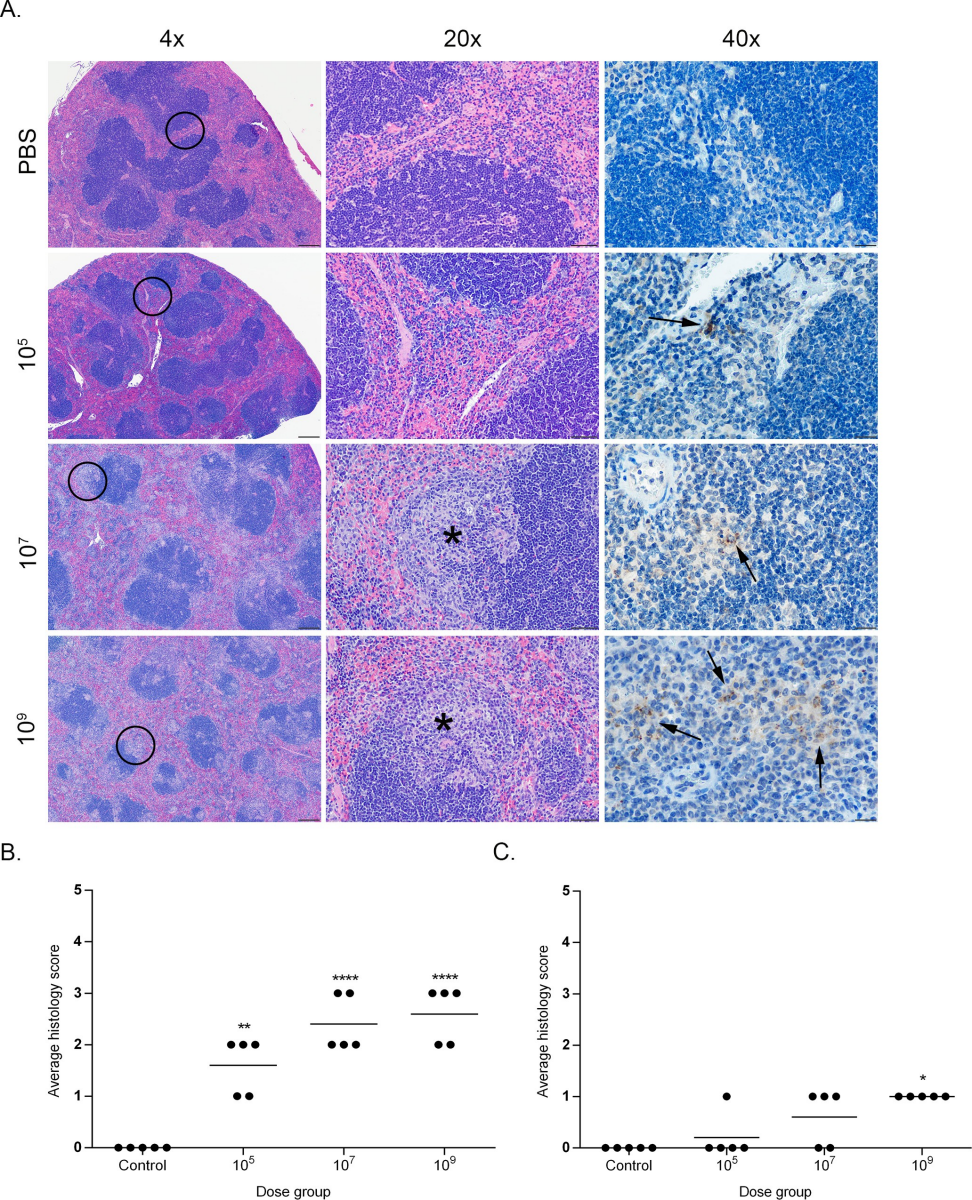
Histopathology of the spleen in mice infected with *B*. *canis*. (A) Representative images of histopathology and immunohistochemistry of the spleen of mice infected intraperitoneally with PBS (top row) or *B*. *canis* RM6/66 at a low dose (10^5^, second row), mid-range dose (10^7^, third row), or high dose (10^9^, bottom row) at 2-weeks post-infection. (B) Sections were scored for severity of histiocytic inflammation from 1–4 (S1 Table) at 2 weeks post-infection, and mean scores were compared using ANOVA followed by Tukey’s multiple comparisons test. (C) Sections were scored using the same system at 12 weeks post-infection. The black circles in the left panel indicate the section highlighted to higher magnification in the middle panel. Infection with *B*. *canis* resulted in infiltration by epithelioid macrophages within the marginal zone and lymphoid follicles (*). *Brucella* antigen was detected within the cytoplasm of macrophages by immunohistochemistry (arrows). Magnification 4x (left column, HE, scale bar = 100 μm), 20x (middle column, HE, scale bar = 50 μm), 40x (right column, IHC with DAB chromagen, bar = 20 μm). Two asterisks, P<0.01, four asterisks, P <0.0001.

**Fig 4:**
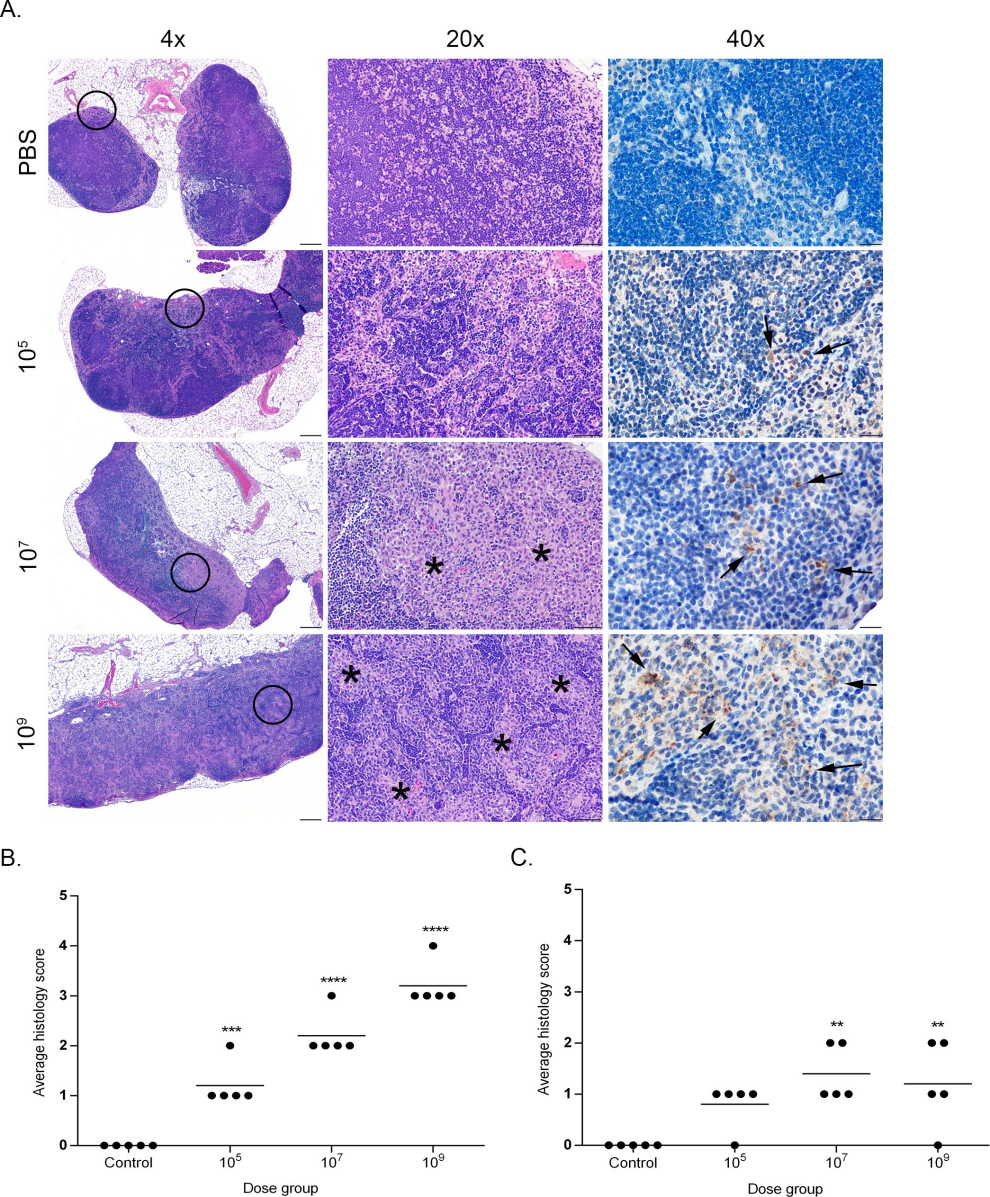
Histopathology of the mesenteric lymph nodes in mice infected with *B*. *canis*. (A) Representative images of histopathology and immunohistochemistry of the mesenteric lymph nodes of mice infected intraperitoneally with PBS (top row) or *B*. *canis* RM6/66 at a low dose (10^5^, second row), mid-range dose (10^7^, third row), or high dose (10^9^, bottom row) at 2-weeks post-infection. (B) Sections were scored for severity of histiocytic inflammation from 1–4 (S1 Table) at 2 weeks post-infection, and mean scores were compared using ANOVA followed by Tukey’s multiple comparisons test. (C) Sections were scored using the same system at 12 weeks post-infection. The black circles in the left panel indicate the section highlighted to higher magnification in the middle panel. Infection with *B*. *canis* resulted in infiltration by epithelioid macrophages within the medullary sinuses zone and surrounding lymphoid follicles (*). *Brucella* antigen was detected within the cytoplasm of macrophages by immunohistochemistry (arrows). Magnification 4x (left column, HE, scale bar = 100 μm), 20x (middle column, HE, scale bar = 50 μm), 40x (right column, IHC with DAB chromagen, bar = 20 μm). Three asterisks, P<0.001, four asterisks, P <0.0001.

Histopathologic changes within the spleen were characterized by aggregates of epithelioid macrophages expanding the marginal zone and infiltrating lymphoid follicles (Fig 3A). The space occupied by these histiocytic infiltrates was significantly higher in the mid to high dose groups (10^7^, 10^9^) than in the low dose (10^5^) and uninfected controls. The level of infiltration also peaked for all groups at 2- to 4-weeks post-infection with no significant differences noted between groups by 6-weeks post-infection. A similar change was noted within the mesenteric lymph nodes in which the medullary sinuses were expanded by varying numbers of epithelioid macrophages (Fig 4A). As in the spleen, the percentage of sinus space occupied by macrophages was significantly higher in the mid and high dose groups (10^7^, 10^9^) and peaked for all groups early in the course of infection at 1–2 weeks. In both the spleen and mesenteric lymph nodes, although the number of infiltrating macrophages declined after 4-weeks post-infection, the infiltrates were never completely cleared in the mid and high dose groups (10^7^, 10^9^).

**Fig 5:**
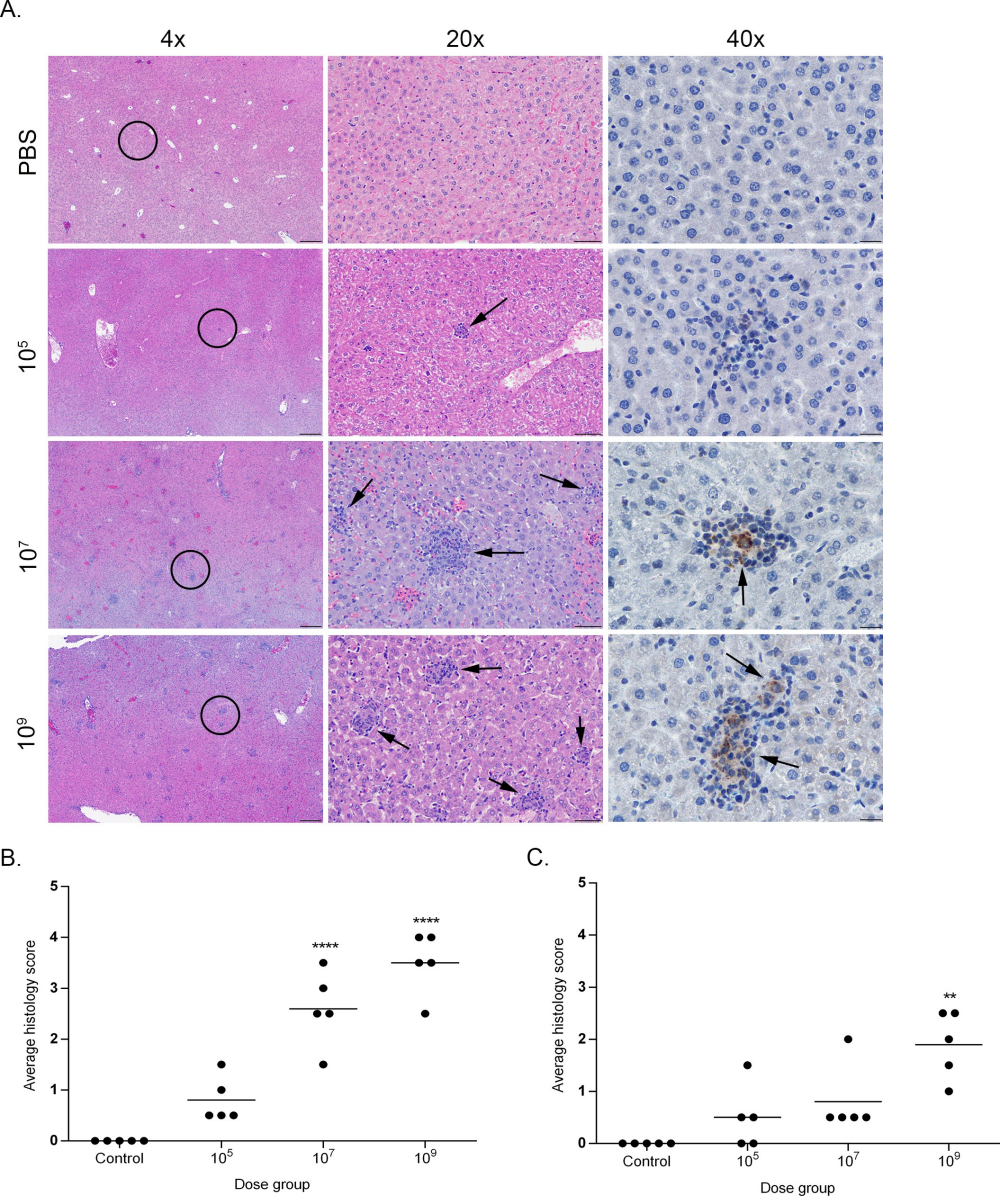
Histopathology of the liver in mice infected with *B*. *canis*. (A) Representative images of histopathology and immunohistochemistry of the liver of mice infected intraperitoneally with PBS (top row) or *B*. *canis* RM6/66 at a low dose (10^5^, second row), mid-range dose (10^7^, third row), or high dose (10^9^, bottom row) at 2-weeks post-infection. (B) Sections were scored for severity of lesions on a scale of 1–4 based on the number of microgranulomas and prevalence of periportal inflammation (S1 Table) at 2 weeks post-infection, and mean scores were compared using ANOVA followed by Tukey’s multiple comparisons test. (C) Sections were scored using the same system at 12 weeks post-infection. The black circles in the left panel indicate the section highlighted to higher magnification in the middle panel. Infection with *B*. *canis* resulted in multifocal, random microgranuloma formation (*) and predominantly mononuclear periportal inflammation. *Brucella* antigen was detected within the cytoplasm of macrophages in the microgranulomas by immunohistochemistry (arrows). Magnification 4x (left column, HE, scale bar = 100 μm), 20x (middle column, HE, scale bar = 50 μm), 40x (right column, IHC with DAB chromagen, bar = 20 μm). Four asterisks, P <0.0001.

The liver exhibited variably sized, randomly distributed microgranulomas composed of nodular accumulations of macrophages and lymphocytes (Fig 5A). Additionally, portal areas were expanded by small to moderate numbers of lymphocytes and macrophages with fewer plasma cells and neutrophils. Similar to the lymphoid organs, lesions within the liver were most severe for all treatment groups at 2 and/or 4-weeks post-infection.

A grading system was developed to characterize histopathologic changes in the spleen, mesenteric lymph nodes, and liver (S1 Table). All sections were graded in a blinded fashion (LS). Calculation of histologic scores for each organ revealed a dose-dependent effect with a significant increase (P<0.0001) in lesion severity based on average histologic score as the dose increased between the uninfected controls and low dose (10^5^) to the mid to high dose (10^7^, 10^9^) groups at 2-weeks post-infection, the peak of severity seen for histopathologic lesions in all organs (Figs 3–5B). After 2 weeks, lesion severity declined until no significant differences were observed between treatment groups by 12 weeks post-infection (Figs 3–5C). Nevertheless, histopathologic scores remained significantly higher than control mice in the two higher dose groups (10^7^, 10^9^) in the liver and mesenteric lymph nodes, and in the high dose group (10^9^) in the spleen.

**Fig 6:**
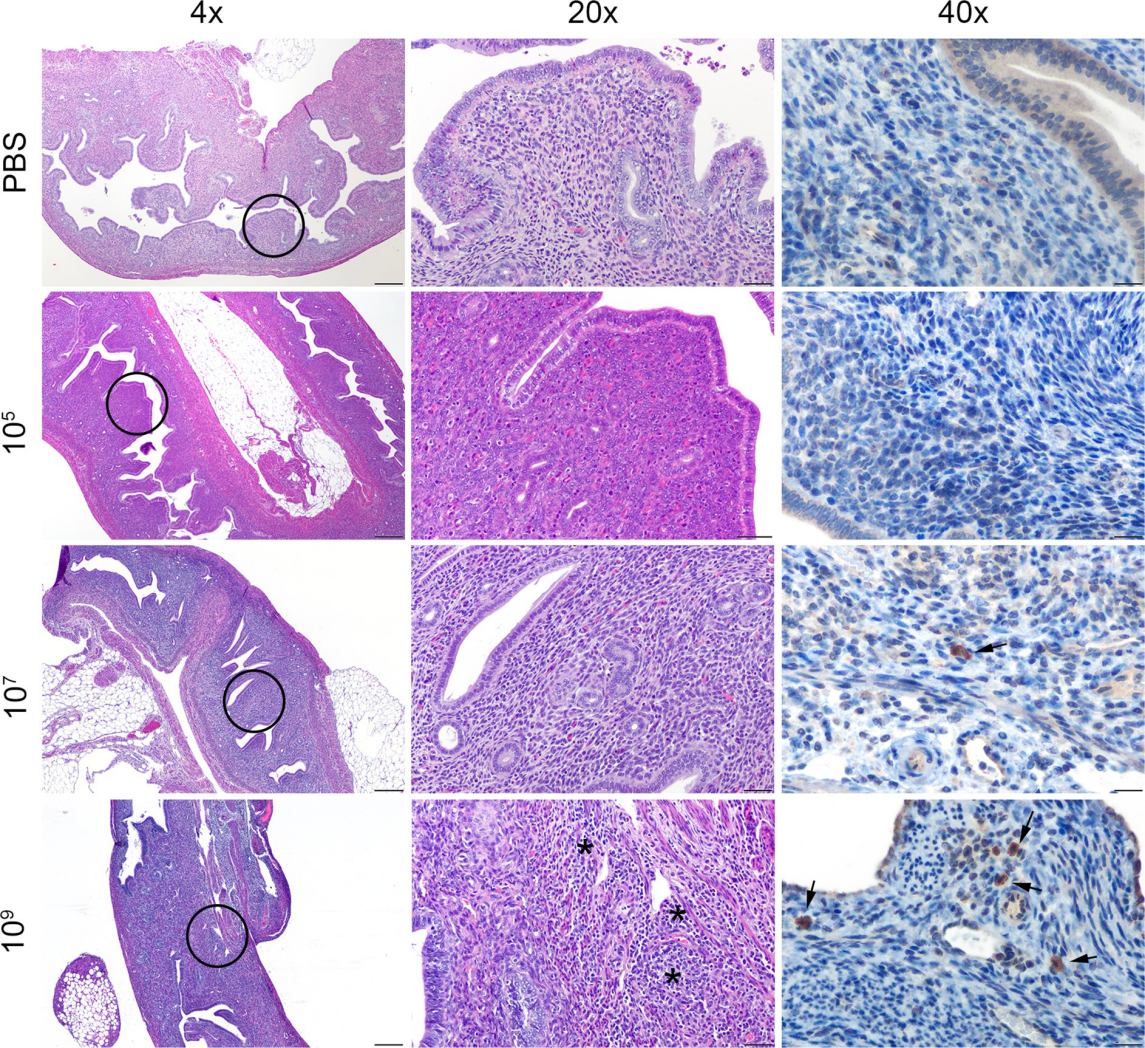
Histopathology of the uterus in mice infected with *B*. *canis*. Representative images of histopathology and immunohistochemistry of the uterus of mice infected intraperitoneally with PBS (top row) or *B*. *canis* RM6/66 at a low dose (10^5^, second row), mid-range dose (10^7^, third row), or high dose (10^9^, bottom row) at 2-weeks post-infection. The black circles in the left panel indicate the section highlighted to higher magnification in the middle panel. Infection with *B*. *canis* resulted in histiocytic, neutrophilic, and lymphocytic peritonitis and metritis (*). *Brucella* antigen was detected within the cytoplasm of macrophages in the outer myometrium by immunohistochemistry (arrows). Magnification 4x (left column, HE, scale bar = 100 μm), 20x (middle column, HE, scale bar = 50 μm), 40x (right column, IHC with DAB chromagen, bar = 20 μm).

Lesions within the uterus were confined to the high dose group (10^9^) and consisted of moderate to marked histiocytic, lymphocytic, and neutrophilic inflammation extending from the serosal surface into the myometrium (Fig 6). Inflammatory lesions occurred alongside a peritonitis composed of the same inflammatory cells accompanied by foci of necrosis in the adipose tissue. Metritis and peritonitis were most severe during 1- and 2-weeks post-infection and then declined to mild inflammation from 4-weeks post-infection until the end of the study. Scattered neutrophils and mononuclear cells were observed within the lamina propria of the endometrium in all groups, including the control group, and no significant inflammation was observed within the endometrium in any group.

To confirm the histologic lesions were directly associated with *Brucella* infection, IHC was performed to colocalize *Brucella* antigen within foci of inflammation. *Brucella* antigen was detected within the cytoplasm of epithelioid macrophages in the spleen and mesenteric lymph nodes, and liver in all dose groups by IHC, further supporting the etiology of the histopathologic changes (Figs 3 and 4A). While antigen was detected within macrophages in microgranulomas in the liver of the mid (10^7^) and high (10^9^) dose groups, no immunopositive signal was detected in the rare microgranulomas from mice in the low dose group (10^5^). *Brucella* antigen was also detected intracellularly within randomly scattered macrophages in the lung, uterus, and mesenteric and perirenal adipose tissue in the mid (10^7^) and high (10^9^) dose groups (S3 Fig).

### Infection induces a *Brucella*-specific IgG humoral immune response

**Fig 7:**
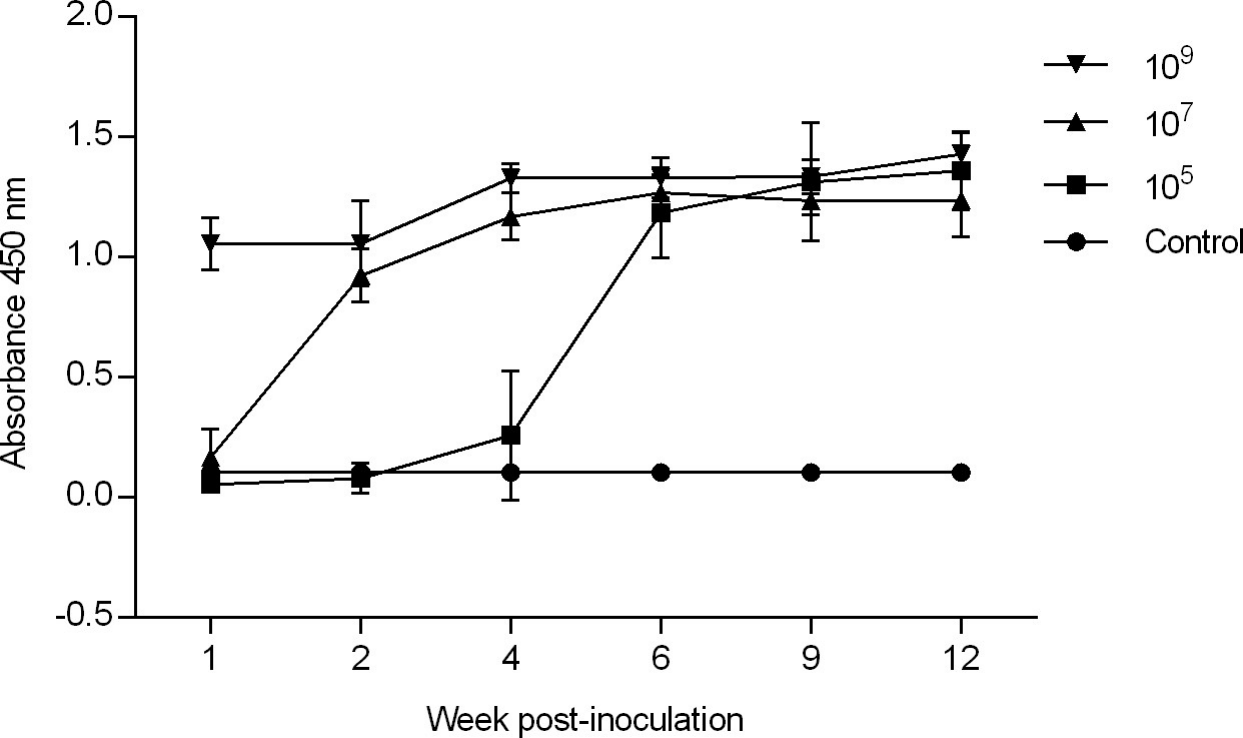
Humoral response to *B*. *canis* infection in mice. Anti-*Brucella* specific IgG indirect ELISA was detected in sera from mice infected intraperitoneally with PBS or *B*. *canis* at doses of 10^5^, 10^7^, and 10^9^ at 1, 2, 4, 6, 9, and 12-weeks post-infection. Mice in all treatment groups developed a significantly higher IgG response compared with the uninfected control group. Total *Brucella*-specific IgG is expressed as the mean absorbance per group plus the standard deviation. Statistical significance was determined by ANOVA followed by Tukey’s multiple comparison of each treatment group (n = 5) to the uninfected controls (n = 6). Three asterisks, P <0.0001.

All mice in all treatment groups developed a significant humoral immune response (anti-*Brucella* specific IgG) to *Brucella canis* infection that varied in time of induction in a dose-dependent manner (Fig 7). Mice in the high dose group (10^9^) developed a marked increase in anti-*Brucella* IgG beginning at 1 week while the same change was noted until 2 and 6 weeks for the mid (10^7^) and low dose (10^5^) groups, respectively. No increase in IgG level was detected in the control group. From 6 weeks until the end of the study at 12 weeks, total IgG response did not significantly differ in mice in any treatment group. Once total IgG had reached a significant elevation above control absorbance values, levels of *Brucella*-specific IgG antibodies maintained at a steady level throughout the remaining study period for all treatment groups (Fig 7).

## Discussion

Over the last several decades, the behavior and pathology of smooth *Brucella* sp in mice has been extensively characterized [16]. Unfortunately, the kinetics of infection of *B*. *canis* in mice is not as well understood. Mice have been heavily utilized and proved valuable in investigation of host-pathogen interactions and vaccine efficacy for smooth *Brucella* species, and have been employed in several recent studies to assess vaccine candidates against *B*. *canis*, demonstrating that they could also fulfill the role as a versatile and economical laboratory model for canine brucellosis [18–21]. The need for an effective laboratory model for *B*. *canis* is evidenced by the recent rise in detection of human cases [9–11], rise in prevalence in stray dog populations [8,22,23], deficiencies in current diagnostic paradigms, and, most importantly, the lack of a protective vaccine. Although numerous similarities exist between smooth and rough *Brucella* sp infection in mice, differences in colonization and induced pathology exist and infection protocols therefore cannot be extrapolated from studies involving smooth species to those involving rough species, such as *B*. *canis*. In an effort to help standardize the mouse as a model for studying *B*. *canis*, we have characterized the kinetics of colonization, pathological lesions, and humoral immune response against infection with this bacterium at multiple doses. Three doses were selected: a low dose of 10^5^ CFU which reflects the level of inoculation typically used for smooth *Brucella* sp [16], a mid-range dose of 10^7^ CFU which has been previously used to infect mice with *B*. *canis* [24], and a high dose of 10^9^ CFU which induces significant morbidity and mortality in the case of smooth *Brucella* sp infection in mice. Importantly, we have established an optimum dose to be used to induce consistent organ colonization and pathology in mice.

The results of this study and few previous studies investigating *B*. *canis* infection in mice indicate that, similar to smooth *Brucella* sp, *B*. *canis* exhibits a tropism for organs of the reticuloendothelial system and is able to persist at these sites for several weeks. As demonstrated by Carmichael et al, *B*. canis at a dose of 10^9^ CFU colonized and induced granulomatous lesions in the spleen of Swiss white mice up to 4 weeks post-infection [17]. Similarly, mice in the mid (10^7^) and high (10^9^) dose groups demonstrated diffuse histiocytic infiltration of the spleen that persisted for 6 weeks post-infection. However, the mice of this study maintained colonization of the spleen longer, through 9 weeks post-infection in the mid dose group (10^7^) and 12 weeks post-infection in the high dose group (10^9^). A more recent study infected BALB/c mice with 10^7^ CFU of a field strain of *B*. *canis* and noted replication 3–6 weeks post-infection in all examined organs [24]. *B*. *canis* was able to persist through 12 weeks but induced minimal splenomegaly and few hepatic granulomas [24]. In contrast, significant replication was not noted in any examined organ at any dose in this study, although it cannot be definitively concluded that *B*. *canis* does not replicate in C57BL/6 mice from the results of a single experiment. Instead, colonization steadily decreased until clearance in all organs was achieved by 12-weeks post-infection, apart from the spleen in the high dose group (10^9^). Such a difference is potentially owed to the difference in mouse strain and/or *B*. *canis* strain utilized, as the previous study employed a field strain, while this study utilized the ATCC reference strain, *B*. *canis* RM 6/66. This factor is a critical consideration for standardization of infection protocols involving *B*. *canis* experiments in mice.

The kinetics of colonization and pathology induced by *B*. *canis* in this study holds key similarities and differences with infection of mice with smooth *Brucella* species. As seen in this study, the most significantly affected organs in mice infected with smooth *Brucella* sp are the liver and spleen with bacterial colonization higher in the spleen than in the liver [16]. Clearance of smooth *Brucella* sp in the liver occurs sooner, as early as 3–4 weeks [11,13], with colonization of the spleen persisting much longer, frequently beyond 36 weeks [25,26]. Similarly, mice in the mid (10^7^) and high (10^9^) dose groups demonstrated higher colonization in the spleen by 2 weeks and colonization persisted longer. Splenomegaly is a prominent feature of infection with smooth *Brucella* sp, in mice as well as humans and natural mammalian hosts [2,16,27]. In mice, splenomegaly peaks from 3–16 weeks and then slowly declines, although splenic weight rarely returns to a normal [16,28]. Similarly, mice in the high dose group (10^9^) developed significant splenomegaly early in the course of infectionbut splenic weight then declined significantly and regained normal weight by 6 weeks. The splenomegaly induced by *B*. *canis* therefore appears to be transient in direct contrast to that induced by smooth species. Additionally, the dose required to induce this change with *B*. *canis* (10^9^ CFU) is notably higher than that required for smooth species, in which a dose of 10^4^ to 10^6^ CFU is commonly used [16].

Early in the course of infection, smooth *Brucella* species replicate in target organs, including the liver and spleen, with peak CFU typically occurring at 2–3 weeks [25,26]. However, the CFU of *B*. *canis* in the mice of this study did not significantly increase over time in any organ, instead demonstrating a slow, progressive decline apart from mice in the high dose group (10^9^) which maintained colonization of the spleen at a steady level of approximately 4 logs. *B*. *canis* has a propensity to establish long-lasting, chronic infections in both humans and dogs, with prolonged incubation periods in which the infected individual may be completely asymptomatic [6,10,11]. This pattern appears to hold true for mice as well.

Mice infected with dose of smooth *Brucella* sp higher than 10^8^ CFU demonstrate obvious clinical signs of disease with death occurring in 50% of mice inoculated with 10^9^ CFU of *B*. *abortus* 2308 within 48 h [29,30]. In contrast, mice in this study showed no signs of illness at doses as high as 10^9^ CFU throughout the entire study period. This fact, in addition to the lack of splenomegaly and significant histologic lesions induced by the standard smooth *Brucella* sp inoculation dose of 10^5^ CFU, demonstrates that *B*. *canis* is less virulent in mice than are smooth *Brucella* sp. Therefore, direct extrapolation of data from mouse studies involving smooth *Brucella* sp cannot be applied to studies involving *B*. *canis*.

Histologic lesions induced by *B*. *canis* in this study resemble those induced by smooth *Brucella* sp. The liver demonstrates accumulation of macrophages within nodular aggregates, or granulomas [30,31]. Multifocal microgranulomas were a prominent finding in the livers of mice in the mid (10^7^) and especially the high (10^9^) dose groups although they were rare and small in mice in the 10^5^ group. As infection in mice with smooth *Brucella* sp progresses, granulomas slowly decrease in the size and number around 5–6 weeks [28], a pattern that was replicated in this study. During the first 2 weeks of smooth *Brucella* sp infection, the spleen is infiltrated by moderate number of macrophages [28,32], as seen in mice in the mid (10^7^) and high (10^9^) dose groups. It therefore appears that *B*. *canis* induces similar histopathologic lesions in the liver and spleen as do smooth *Brucella* specied but it is again critical to note that the dose required to do so (10^7^−10^9^ CFU) is notably higher than that required for smooth species (10^4^−10^6^

The pathology described in this study also shows similarities to that induced by *B*. *canis* in the natural canine host. While most commonly recognized as a cause of reproductive disease in dogs, *B*. *canis* frequently causes lesions in organs of the reticuloendothelial system as smooth *Brucella* sp do. A commonly recognized sign is regional lymphadenopathy with lymph nodes exhibiting a combination of lymphoid hyperplasia and infiltration of the sinuses by neutrophils and macrophages [17]. Infiltration of the mesenteric lymph nodes by macrophages was a prominent feature in mice in mid (10^7^) and high (10^9^) dose groups that persisted through 6 weeks. Livers of infected dogs may also show small accumulations of macrophages, a feature that was present within the liver of mice in all dose groups [17]. Given the propensity of *B*. *canis* to colonize the lymph nodes of dogs and the consistent histologic lesions detected in mice of this study, the lymph nodes appear to be an important organ to evaluate in *B*. *canis* studies in mice.

*Brucella* spp. are classically recognized as pathogens of the pregnant uterus and commonly cause a range of reproductive complications in animals and humans [2,33]. Studies of reproductive pathology in brucellosis have predominantly focused on the effects of infection on the pregnant uterus, in which smooth *Brucella* sp will establish a persistent and productive infection [2]. Recently, tropism of smooth *Brucella* sp for the nongravid uterus has been demonstrated in guinea pigs [34]. Colonization of the nongravid uterus was noted in the mice of this study at all dose groups and small numbers of macrophages within the myometrium and uterine serosa demonstrated immunopositivity for *Brucella* antigen. Significant peritonitis and metritis were observed in the high dose group (10^9^), secondary to intraperitoneal inoculation, and it is likely that the myometrial inflammation resulted from extension from the inflamed peritoneum. Alternatively, uterine colonization in this study may reflect bacteremic distribution of *B*. *canis* as is suspected to be the case for the low levels of colonization in the lung and kidney. Nevertheless, colonization was higher in the two higher dose groups (10^7,^10^9^) at 1-week post-infection than in the lung and kidney (Fig 1C, 1E and 1F], which may reflect some degree of tropism. The possibility of tropism could be further investigated using alternative means of inoculation, such as intra-tracheal, to avoid the possibility of local extension from the peritoneum. Additionally, the colonization of *B*. *canis* within the reproductive organs of a pregnant female has not been investigated in any laboratory rodent model and the potential for mice to serve as models to investigate the reproductive consequences of *B*. *canis* infection remains to be determined.

Mice infected with smooth *Brucella* sp develop a detectable anti-*Brucella* antibody level around 2 weeks post-infection, after which antibody levels remain high [16,35]. Similarly, *B*. *canis* induced a significant increase in anti-*Brucella* IgG in mice in all dose groups that persisted at a steady level to the end of the study, even beyond the time of clearance of the organism from the examined organs. This coincides with the antibody response in dogs which levels persisting for over 4 years and as long as 6 months after bacteremia ceases [36]. Interestingly, the timing of the induction of a humoral response appeared to be dose related, with mice in the high dose (10^9^) group developing a significant increase in anti-*Brucella* IgG by 1 week while mice did not develop a comparable response until 2 and 6 weeks in the mid (10^7^) and low (10^5^) dose groups, respectively.

Strain of mouse utilized in brucellosis studies has long been a controversial topic. C57BL/6 mice have historically been perceived as being more resistant to *Brucella* infection than the more “susceptible” DBA2, C2H/He, and BALB/c strains owing to the TH1 polarization of their immune response [32,37]. While clearance is achieved earlier in “resistant” strains such as C57BL/6, the number of smooth *Brucella* colonizing the spleen at the onset of infection and the replication profile is similar across strains. This pattern appears to apply to *B*. *canis* infection in mice as well. Mice in the 10^7^ group compared to BALB/c mice infected with 10^7^ CFU demonstrated a similar level of colonization in the spleen during the first 4 weeks of infection with CFU averaging between 4 and 5 logs [24]. However, BALB/c mice maintained colonization through 12 weeks while clearance occurred in the C57BL/6 mice by 9 weeks. The perceived “resistance” to infection in C57BL/6 mice should not be viewed as a deterring factor and, in fact, C57BL/6 mice offer many advantages in studying brucellosis. Importantly, the C57BL/6 strain is the most commonly utilized background strain for development of genetic knockouts and has been shown to have highly similar host-pathogen interaction mechanisms previously described in humans and domestic animals [38,39]. C57BL/6 could serve similarly as a valuable model for studying the pathogenesis of canine brucellosis.

## Conclusions

This study deepens the knowledge of *B*. *canis* infection in mice and identifies multiple similarities to infection with smooth *Brucella* sp and to natural infection in dogs. The mouse is susceptible to systemic infection with *B*. *canis* and could serve as a useful model to investigate host-pathogen interactions and vaccine candidates as it does for smooth *Brucella* species. Mice in the 10^7^ and 10^9^ groups developed consistent histopathologic lesions of infection and persistent colonization of reticuloendothelial organs through 6 weeks. A dose of 10^7^ therefore appears to be a suitable inoculation dose when studying *B*. *canis* in mice, especially in regards to vaccine efficacy studies, in contrast to the standard challenge dose of 2x10^5^ CFU used with smooth *Brucella* species. The potential for mice to serve as a model to investigate the reproductive pathogenesis of *B*. *canis* needs to be further investigated utilizing alternative inoculation methods and pregnant females. Most importantly, the behavior of a standard reference strain of *B*. *canis* in mice has been standardized, allowing for further vaccine efficacy studies to proceed.

## Supporting information

S1 Fig Liver weight in mice inoculated with *B. canis* RM6/66 or PBS over 12 weeks. Hepatomegaly was induced by a high dose (10^9^) of *B*. *canis* at 1-week post-infection (A). Afterwards, liver weight declined with no significant differences noted between groups by 2-weeks post-infection. Data points represent the mean liver weight plus the standard deviation for all animals in each dose group at each time point. Mean liver weight from each dose group (n = 5) was compared at each time point to mean liver weight of the uninfected control mice (n = 6) and statistical significance was determined by ANOVA followed by Tukey’s multiple comparison test. Three asterisks, P <0.00001.
(TIF)

S2 Fig Macroscopic appearance of the liver in mice inoculated with *B. canis* RM6/66 at 2 weeks. Macroscopic changes were induced by a high dose group (10^9^) of *B*. canis at 2-weeks post-infection. The liver in animals in the control group (A) appeared homogeneously tan while animals in the high dose (10^9^) group (B) demonstrated loss of the homogeneous appearance and replacement by multifocal zones of pallor surrounded by a thin hyperemic zone.
(TIF)

S3 Fig Immunohistochemistry to anti-*Brucella* antigen in mice inoculated with *B. canis* RM6/66 at 2 weeks. Following inoculation with a dose of 10^9^ CFU, *Brucella* antigen was detected within macrophages (arrows) scattered throughout the myometrium (A), perirenal adipose tissue (B), alveolar septa (C), and mesenteric adipose tissue (D). Magnification 40x, HE, scale bar = 20 μm.
(TIF)

S1 Table Histopathologic grading system for *B. canis* infection in mice. Spleen, mesenteric lymph nodes, and liver were evaluated for type and severity of inflammation and graded on a scale of 0 to 4.
(DOCX)

S2 Table Percentage of mice exhibiting colonization of *B. canis* RM6/66 over 12 weeks. Animals were divided into 3 treatment groups and inoculated intraperitoneally with a low (10^5^), mid (10^7^), or high (10^9^) dose of *B*. *canis*. Five animals from each group were euthanized at 1-, 2-, 4-, 6-, 9-, and 12-weeks post-infection. Colonization was evaluated in the liver, spleen, uterus, bone marrow, lung, and kidney.
(DOCX)
