# Supplementary figures and images for: Characterization of Brucella canis infection in mice
Source: PLoS One. 2019 Jun 20;14(6):e0218809. doi: 10.1371/journal.pone.0218809 (PMC6586350; doi:10.1371/journal.pone.0218809)

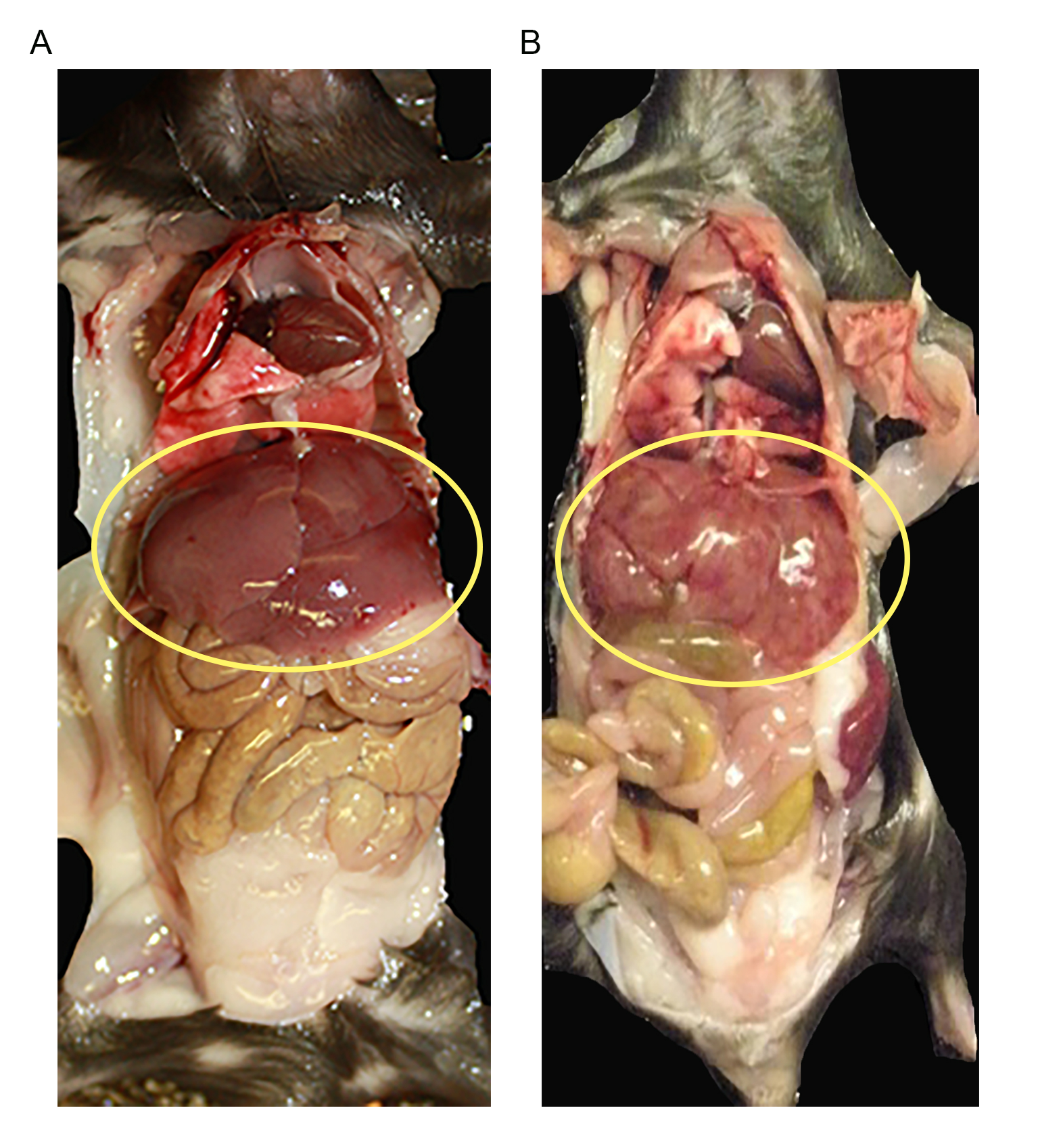

Supplement: S2 Fig — Macroscopic changes were induced by a high dose group (109) of B. canis at 2-weeks post-infection. The liver in animals in the control group (A) appeared homogeneously tan while animals in the high dose (109) group (B) demonstrated loss of the homogeneous appearance and replacement by multifocal zones of pallor surrounded by a thin hyperemic zone. (TIF) [file pone.0218809.s002.tif]

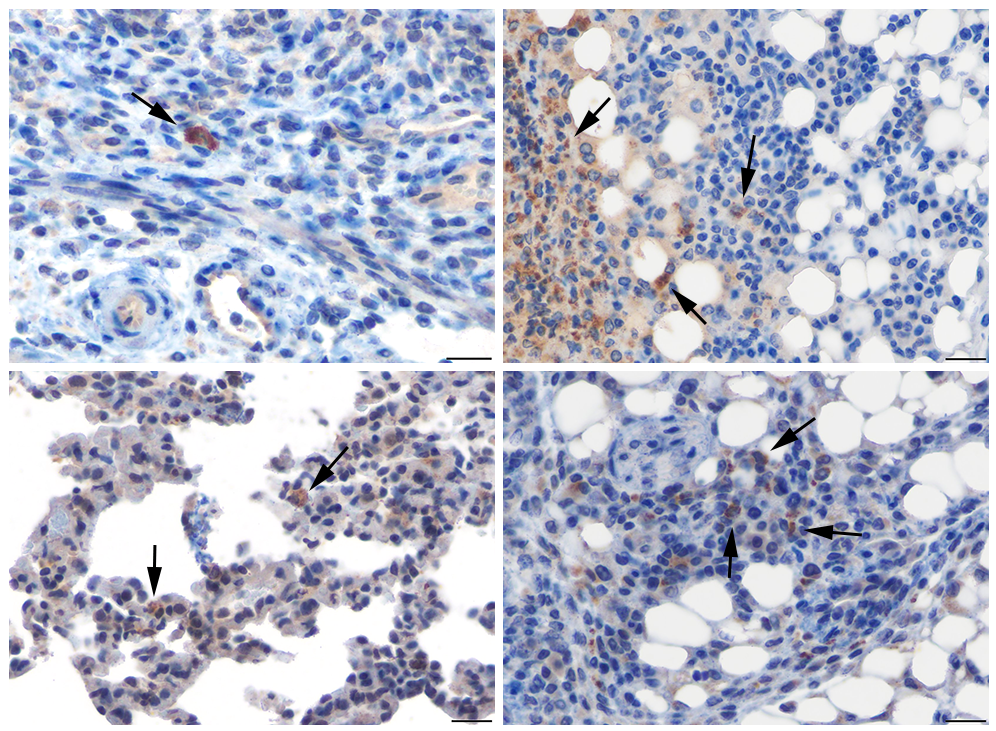

Supplement: S3 Fig — Following inoculation with a dose of 109 CFU, Brucella antigen was detected within macrophages (arrows) scattered throughout the myometrium (A), perirenal adipose tissue (B), alveolar septa (C), and mesenteric adipose tissue (D). Magnification 40x, HE, scale bar = 20 μm. (TIF) [file pone.0218809.s003.tif]
