# Supplementary material for: Characterization of Brucella canis infection in mice
Source: PLoS One. 2019 Jun 20;14(6):e0218809. doi: 10.1371/journal.pone.0218809 (PMC6586350; doi:10.1371/journal.pone.0218809)
Supplement: S1 Table — Spleen, mesenteric lymph nodes, and liver were evaluated for type and severity of inflammation and graded on a scale of 0 to 4. (DOCX) [file pone.0218809.s004.docx]

**Table S1: Histologic grading system for *B. canis* infection in mice**

Liver

| Lesion | Score | Description |
| --- | --- | --- |
| Periportal inflammation | 0 | None |
|  | 1 | Minimal- <25% affected |
|  | 2 | Mild- 25%-50% |
|  | 3 | Moderate- 51%-75% affected |
|  | 4 | Marked- 100% affected |
| Microgranulomas | 0 | None |
|  | 1 | Minimal- one focus per ten 10x objectives |
|  | 2 | Mild- two to four foci per ten 10x objectives |
|  | 3 | Moderate- five to 10 foci per ten 10x objectives |
|  | 4 | Marked- >10 per ten 10x objectives |

Spleen

| Lesion | Score | Description |
| --- | --- | --- |
| Histiocytic inflammation | 0 | None |
|  | 1 | Minimal- filling <25% of marginal zone |
|  | 2 | Mild- filling 25-50% of marginal zone |
|  | 3 | Moderate- filling 51-75% of marginal zone |
|  | 4 | Marked- filling >75% of marginal zone |

Mesenteric lymph nodes

| Lesion | Score | Description |
| --- | --- | --- |
| Histiocytic inflammation | 0 | None |
|  | 1 | Minimal- filling <25% of sinus space |
|  | 2 | Mild- filling 25-50% of sinus space |
|  | 3 | Moderate- filling 51-75% of sinus space |
|  | 4 | Marked- filling >75% of sinus space |
