# Supplementary material for: Characterization of Brucella canis infection in mice
Source: PLoS One. 2019 Jun 20;14(6):e0218809. doi: 10.1371/journal.pone.0218809 (PMC6586350; doi:10.1371/journal.pone.0218809)
Supplement: S2 Table — Animals were divided into 3 treatment groups and inoculated intraperitoneally with a low (105), mid (107), or high (109) dose of B. canis. Five animals from each group were euthanized at 1-, 2-, 4-, 6-, 9-, and 12-weeks post-infection. Colonization was evaluated in the liver, spleen, uterus, bone marrow, lung, and kidney. (DOCX) [file pone.0218809.s005.docx]

**Table S2: Percentage of mice exhibiting colonization of *B. canis* RM6/66 over 12 weeks**

| Week | Dose group | Liver | Spleen | Uterus | Bone marrow | Lung | Kidney |
| --- | --- | --- | --- | --- | --- | --- | --- |
| 1 | 10^5^ CFU | 0% | 20% | 0% | 0% | 0% | 0% |
| 1 | 10^7^ CFU | 100% | 100% | 100% | 100% | 100% | 100% |
| 1 | 10^9^ CFU | 100% | 100% | 100% | 100% | 100% | 100% |
| 2 | 10^5^ CFU | 60% | 80% | 0% | 0% | 80% | 0% |
| 2 | 10^7^ CFU | 100% | 100% | 100% | 40% | 100% | 100% |
| 2 | 10^9^ CFU | 100% | 100% | 100% | 80% | 100% | 100% |
| 4 | 10^5^ CFU | 20% | 20% | 20% | 20% | 20% | 20% |
| 4 | 10^7^ CFU | 100% | 100% | 40% | 80% | 60% | 60% |
| 4 | 10^9^ CFU | 100% | 100% | 100% | 80% | 100% | 80% |
| 6 | 10^5^ CFU | 40% | 100% | 0% | 20% | 60% | 0% |
| 6 | 10^7^ CFU | 40% | 20% | 20% | 0% | 20% | 0% |
| 6 | 10^9^ CFU | 80% | 100% | 60% | 20% | 20% | 0% |
| 9 | 10^5^ CFU | 0% | 0% | 0% | 0% | 0% | 0% |
| 9 | 10^7^ CFU | 20% | 60% | 0% | 0% | 20% | 0% |
| 9 | 10^9^ CFU | 80% | 80% | 0% | 0% | 0% | 0% |
| 12 | 10^5^ CFU | 0% | 0% | 0% | 0% | 0% | 0% |
| 12 | 10^7^ CFU | 20% | 0% | 0% | 0% | 0% | 0% |
| 12 | 10^9^ CFU | 0% | 100% | 0% | 0% | 0% | 20% |
